# A neural network based computational model to predict the output power of different types of photovoltaic cells

**DOI:** 10.1371/journal.pone.0184561

**Published:** 2017-09-12

**Authors:** WenBo Xiao, Gina Nazario, HuaMing Wu, HuaMing Zhang, Feng Cheng

**Affiliations:** 1 Jiangxi Engineering Laboratory for Optoelectronics Testing Technology, Nanchang Hangkong University, Nanchang, China; 2 Department of Pharmaceutical Science, College of Pharmacy, University of South Florida, Tampa, FL, United States of America; Chongqing University, CHINA

## Abstract

In this article, we introduced an artificial neural network (ANN) based computational model to predict the output power of three types of photovoltaic cells, mono-crystalline (mono-), multi-crystalline (multi-), and amorphous (amor-) crystalline. The prediction results are very close to the experimental data, and were also influenced by numbers of hidden neurons. The order of the solar generation power output influenced by the external conditions from smallest to biggest is: multi-, mono-, and amor- crystalline silicon cells. In addition, the dependences of power prediction on the number of hidden neurons were studied. For multi- and amorphous crystalline cell, three or four hidden layer units resulted in the high correlation coefficient and low MSEs. For mono-crystalline cell, the best results were achieved at the hidden layer unit of 8.

## Introduction

The renewable energy sources, such as photovoltaic (PV) cell power generation [1], will become important in the future [2], as it has not only a great potential to solve the current energy crisis but also is environment-friendly to solve the current environmental crisis [3]. The output power of PV cells depends on the solar radiation intensity, device material and device temperature [4] and so on. For example, mono-crystalline, multi-crystalline, and amorphous crystalline silicon solar PV cells exhibit different characteristics in the external work conditions. So, for potential cost savings of the PV power supply, a good prediction model of home power demand and PV power supply is the essential [5, 6, 7, 8 and 9]. In all predicting methods, the artificial neural network (ANN) method has received a considerable amount of attention for power prediction [10]. This is because the ANN methods are used to model complex nonlinear dynamic systems with great success. Specifically speaking, ANN-models do not require the use of specific analytic formulations and physics-based derivations [11], do not need an extensive amount of parameters or complicated calculations [12, 13], and perform better than polynomial regression and multiple linear regression models [14] when modeling a nonlinear system. Traditionally, the system dynamics can be emulated by feeding a measured database into the configured network to train the ANN neurons until either an acceptable precision or the maximum iteration number is reached. In all ANN-models, it is found that the size of the hidden layer neuron is an important parameter [15, 16]. The prediction performance of ANN depends on the selection size of the hidden layer. An underestimated amount of neurons can lead to poor approximation and generalization capabilities, while the excessive nodes could result in over fitting and eventually make the search for the global optimum more difficult. In fact, the number of neurons in the hidden layers is very hard to determine, since there is no ideal analytical formula to represent [17, 18, 19 and 20]. Therefore, some rule-of-thumb methods are proposed to find the correct number of neurons. For example, Camargo et al [21] provided a criterion for the choice of the number of neurons in the hidden layer, which is based on polynomial interpolation theory. Kolmogorov's theorem [22] indicated that the network has only one hidden layer with exactly 2*n* + 1 node, where *n* is the number of input layers. Yuan *et al*. [23] found that the number of hidden neurons can be decided based on information entropy theory. In addition, in order to specify the scope of the number of hidden neurons, the lower and upper boundaries on the number of hidden neurons was given by Jiang *et al*. [24] and Huang *et al*. [25]. They found that the lower boundary can accelerate the learning speed, and the upper bound gives the stopping condition of constructive learning algorithms. In order to further study the PV Power characteristics and evaluate the effects of number of hidden neurons on different types of PV cells’ power prediction based on ANN, the relevant researches were done by using random sampling studies. The optimal values of hidden layer units for these types of PV cells were decided.

In this article, an artificial neural network (ANN) based computational model was introduced to predict the output power of three types of photovoltaic cells, mono-crystalline (mono-), multi-crystalline (multi-), and amorphous (amor-) crystalline. The prediction results were evaluated in terms of correlation coefficient (r) and mean of square error (MSE). The prediction results are very close to the experimental data, and were also influenced by numbers of hidden neurons. The order of the solar generation power output influenced by the external conditions from smallest to biggest is: multi-, mono-, and amor- crystalline silicon cells. In addition, the dependences of power prediction on the number of hidden neurons were studied. For multi- and amorphous crystalline cell, three or four hidden layer units resulted in the high correlation coefficient and low MSEs. For mono-crystalline cell, the best results were achieved at the hidden layer unit of 8.

## Material and methods

### Experimental samples and setup

The experimental samples were 2.8 cm × 2.5 cm mono-crystalline, multi-crystalline and amorphous crystalline PV cells manufactured by QS Solar Company. The PV testing system (number SAC made by Chengdu ZKY Instrument Co., Ltd) was used for data acquisition. The simulated sunlight was produced by Xenon light source. The intensity of the incident light could be adjusted by a six tranches toggle switch. The light intensities of the 1st, 2nd, 3rd, 4th, 5th and 6th tranche correspond to about 600, 700, 800, 900, 1000 and 1100 W/m^2^, respectively. The experimental sample was placed in a temperature control room that was primarily composed of a semiconductor refrigeration device. The current-voltage curves and maximum output powers (MOP) of the PV cells were measured under -10, -5, 0, 5, 10, 15, 20, 25, 27, 30, 35 and 40°C, respectively. Therefore, there are twelve measurement conditions for each light intensity tranches. For example, the measured conditions of light intensity and temperature are, 6th tranche and 40°C, 6th tranche and 35°C, 6th tranche and 30°C, 6th tranche and 27°C, 6th tranche and 25°C, 6th tranche and 20°C, 6th tranche and 15°C, 6th tranche and 10°C, 6th tranche and 5°C, 6th tranche and 0°C, 6th tranche and -5°C, and 6th tranche and -10°C for the 6th tranche, respectively. Then, 72 experimental data points were totally collected, 62 data points among them were chosen to train the neuron network and the remaining 10 data points were used to validate the prediction results.

### Neuron network structure and settings

**Fig 1:**
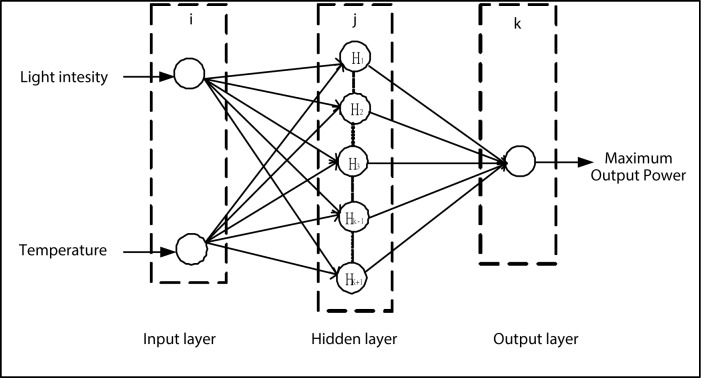
The schematic diagram of network structure.

The structure of the proposed neural network consists of three layers: input, hidden, and output layer as shown in **Fig 1**. The input layer has two nodes (the incident light intensity and device temperature). The output layer has one node (the MOP of PV cells). The input neurons receive data of light intensity and temperature, the hidden neurons receive signals from neurons in the preceding layer, and the output neurons send prediction information to the external part. Two MATLAB functions, Tansig and Purelin, were chosen as the transfer functions in the hidden and output layer, respectively. A MATLAB function, Traingdm, was selected for model training.

## Results and discussion

### Electrical characteristics of PV cells

**Fig 2:**
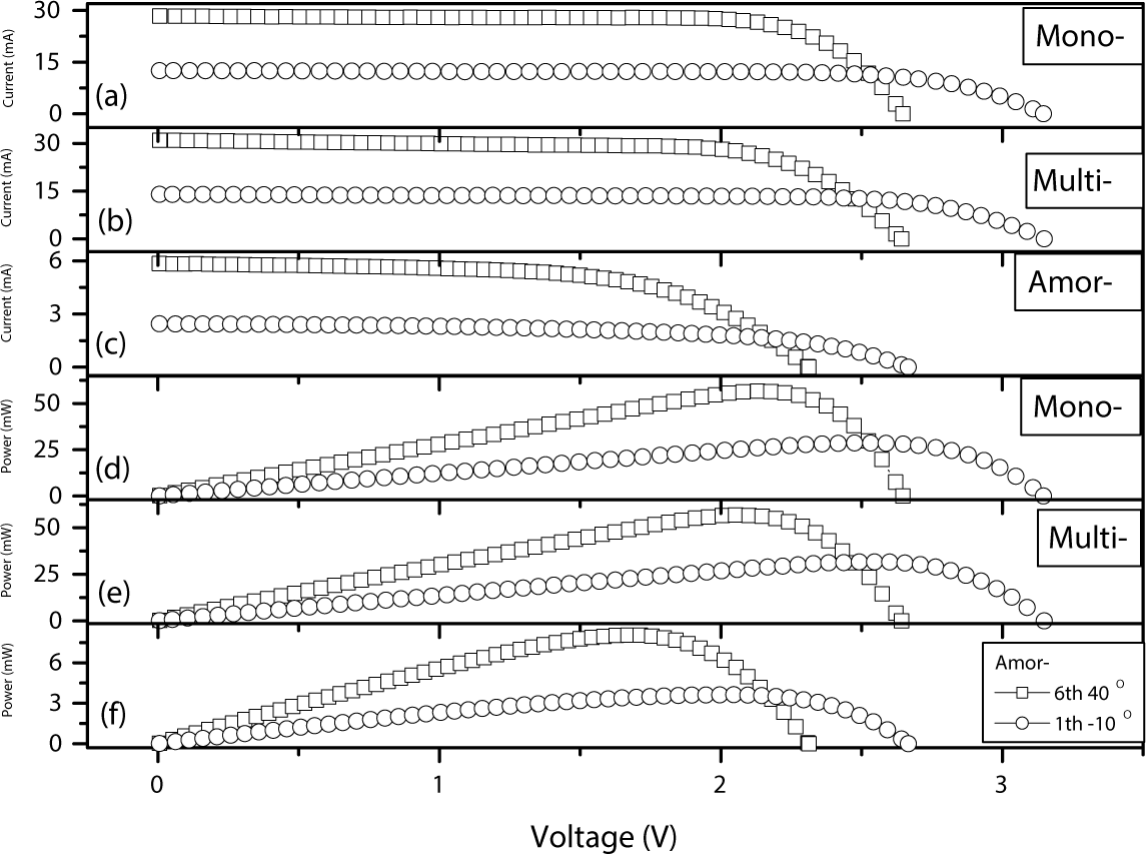
*I-V* and *P-V* curves of mono-crystalline (**a, d**), multi-crystalline (**b, e**) and amorphous crystalline (**c, f**) silicon PV cells measured at two extreme conditions: the 1st tranche (light intensity) and -10°C (temperature), as well as 6th tranche and 40°C.

**Fig 2** shows *I-V* and *P-V* curves of mono-crystalline (a, d), multi-crystalline (b, e) and amorphous crystalline (c, f) silicon PV cells measured at two extreme conditions: the lowest light intensity and temperature (the 1st tranche (light intensity) and -10°C (temperature)), as well as the highest light intensity and temperature (6th tranche and 40°C), respectively. The short-circuit current value changes from 28.370 mA (at the 6th tranche and 40°C) to 12.526 mA (at the 1st tranche and -10°C) for mono-crystalline, 30.960 mA to 14.003 mA for multi-crystalline, and 5.844 mA to 2.449 mA for amorphous crystalline. The open-circuit voltage of changes from 2.647 V to 3.146 V for mono-crystalline, 2.642 V to 3.149 V for multi-crystalline, and 2.309 V to 2.666 V for amorphous crystalline. The relative changes of short-circuit current are approximately 55.8%, 54.8%, and 58.1% for mono-crystalline, multi-crystalline and amorphous crystalline cells, respectively. The relative changes of open-circuit voltage of three types of crystalline cells are approximately 15.9%, 16.1%, and 13.4%. The light intensity and device temperature affect the short-circuit current more than the open-circuit voltage. The findings are consistent with previous studies showing that the short-circuit current is directly proportional to the effective radiation intensity [26], and exhibits a positive temperature coefficient [27]. However, the open-circuit voltage is mainly determined by the band gap of the solar cell material [28].

In addition, the MOP of mono-crystalline, multi-crystalline and amorphous crystalline cells changes from 56.6793 mW to 28.6407 mW, 56.7993 mW to 31.5958 mW, and 8.0477 mW to 3.6296 mW, respectively. The relative changes are approximately 49.5%, 44.4%, and 54.9%. The MOP of amorphous crystalline cell is far less than that of mono- or multi- crystalline cells at the same condition. Amorphous thin-film solar cells are less susceptible to light intensity and cell temperature and have lower efficiency rates because the silicon material of amorphous crystalline cell is not structured or crystalized on a molecular level.

### Predicting results under different numbers of hidden neurons using in the algorithm

**Fig 3:**
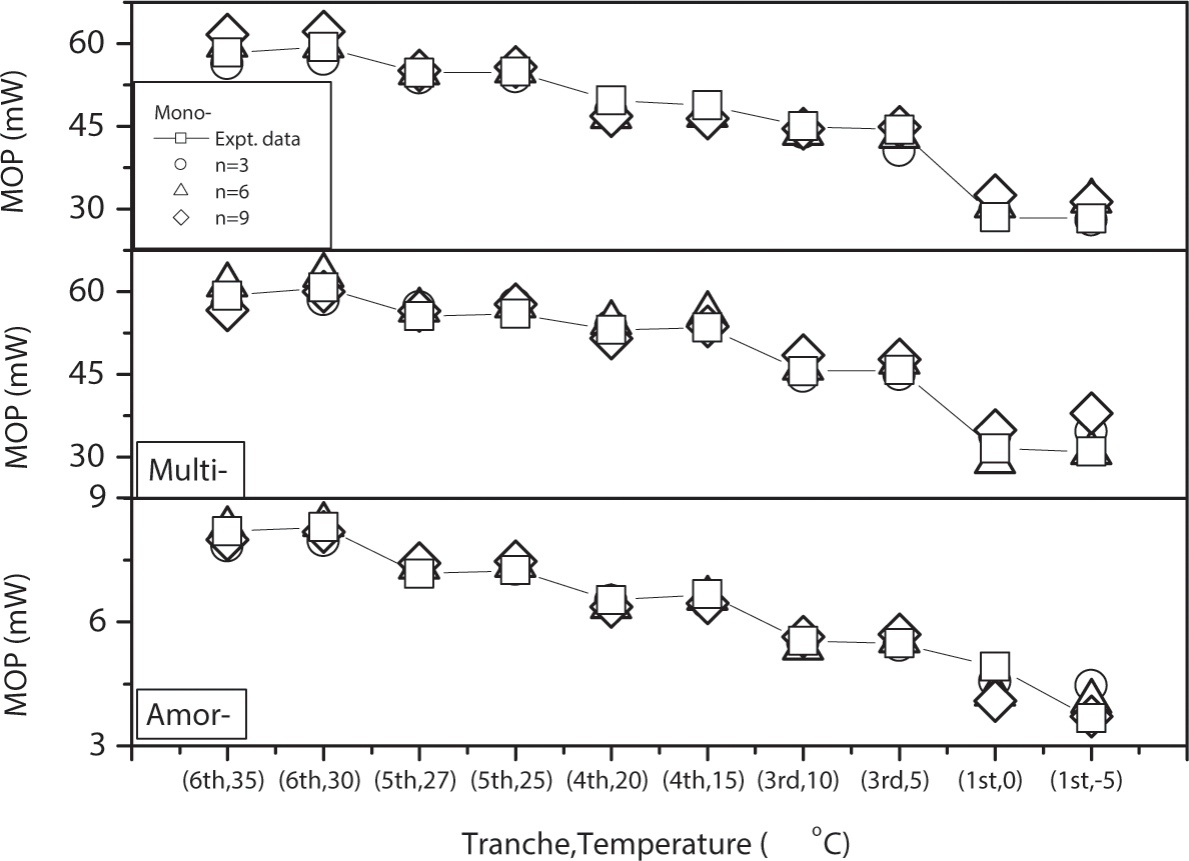
Mono-crystalline (a), multi-crystalline (b) and amorphous crystalline (c) cells compared to experimental data using different numbers of hidden neurons (n = 3, 6, and 9).

**Fig 3** shows the MOP prediction results of mono-crystalline (a), multi-crystalline (b) and amorphous crystalline (c) cells compared to experimental data using different numbers of hidden neurons (n = 3, 6, and 9) in the Neuron Network algorithm. The prediction results are very close to the experimental data, and are also influenced by numbers of hidden neurons, especially for crystalline cells at lower light intensity and temperature.

**Fig 4:**
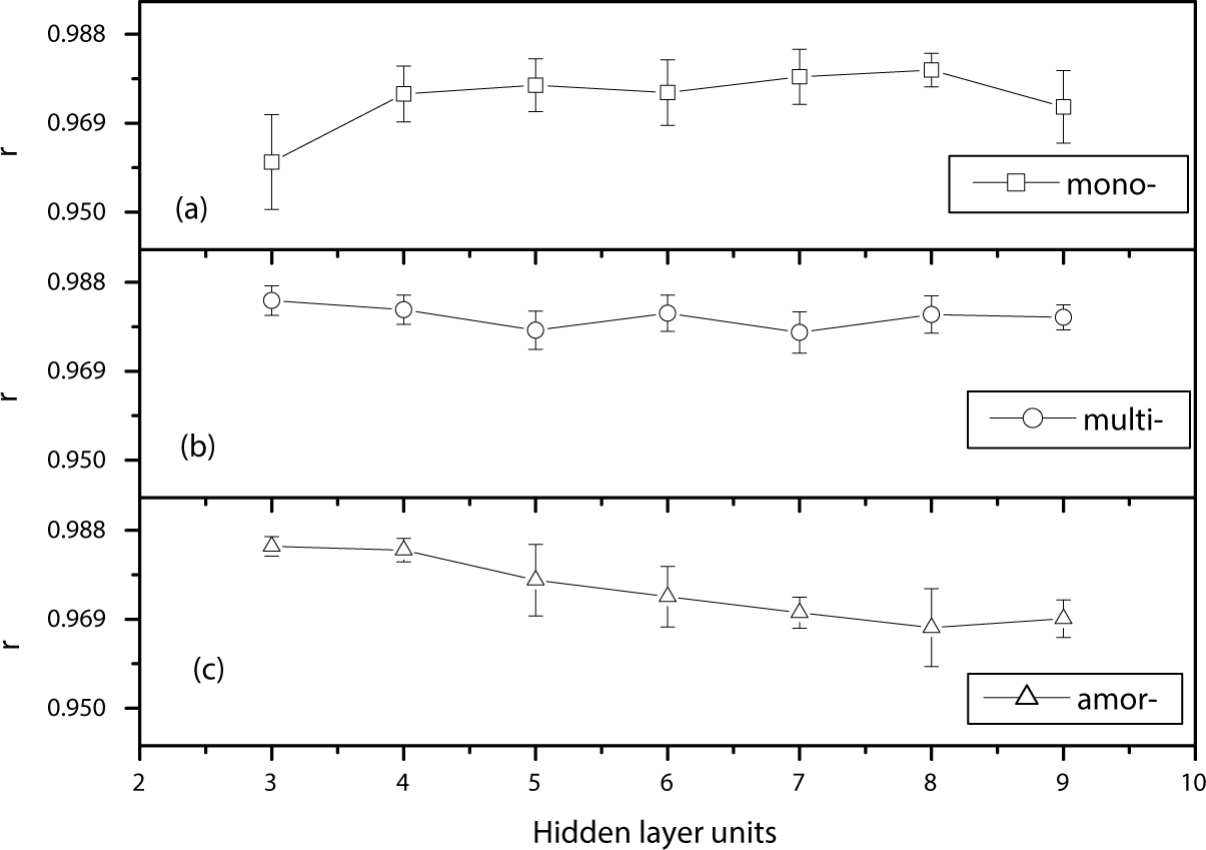
The average correlation coefficients at the different number of hidden layer units in the neuron network algorithm.

**Fig 5:**
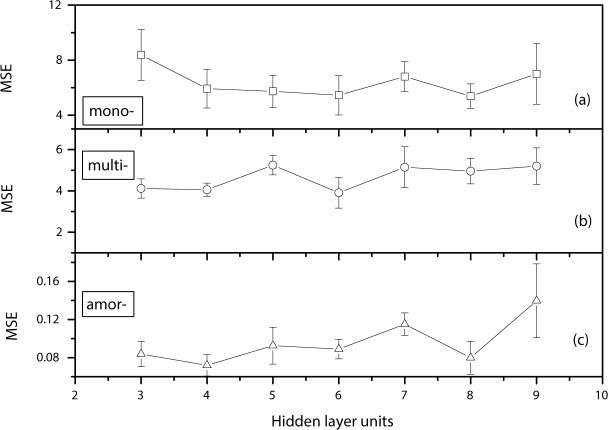
The average MSEs varying with the number of hidden units for mono-(a), multi-(b) and amor-(c) cells.

In order to understand the effect of the number of hidden neurons on the prediction accuracy, ten-fold cross-validations were performed at different numbers of hidden neurons (from 3 to 9). The average correlation coefficients (shown in **Fig 4**), and the average MSEs between experimental and prediction results (**Fig 5**) were calculated. As shown in **Fig 4**, the average value of the correlation coefficient is greater than 0.95 for all three types of cells using any hidden layer numbers, which indicates that there is almost a perfect positive linear relationship between them. Furthermore, those results prove the accuracy and reliabilities of the neuron network algorithm. In addition, it can be seen that the prediction results are affected by the number of hidden layer units in the algorithm. The curve of the average correlation coefficients VS hidden layer units for mono-cells is gradually lifted upwards, then approaches the saturation level and finally drops down. The curve for multi- cells almost doesn't change within those numbers of hidden neurons. However, the curve for amor- cells is significantly decreasing with the numbers of hidden neurons increasing. Therefore, it can be concluded that the relationship between prediction MOP results and the number of hidden units is not a simple function. Finally, it can be seen that, for multi- and amorphous crystalline cell, three hidden layer units gave the highest correlation coefficient. For mono-crystalline cell, the best results were achieved at the hidden layer unit of 8.

The average MSEs between experimental and prediction results for all three types of cells were shown in **Fig 5**. It can be seen that the average MSEs between experimental and prediction results nonlinearly change with the hidden layer units for mono-, multi- and amor- cells. Those results further prove that relationship between prediction MOP results and the number of hidden units is not a simple function. In addition, similar to **Fig 4**, three or four hidden layer neuron units gave the low MSEs for multi- and amorphous crystalline cell. For mono-crystalline cell, the lowest MSEs are achieved at the hidden layer unit of 8. Therefore, the results show that there is no a universal principle (such as Kolmogorov's theorem [20]) to select the number of neurons in the hidden layers for all types of silicon photovoltaic devices.

## Conclusion

In this paper, we built a neural network based computational model to predict the output power of three types of photovoltaic cells. The effects of the number of hidden neurons on the prediction of the output power of mono-, multi- and amorphous crystalline silicon cells were also investigated. Firstly, the order of the solar generation power changes influenced by the external conditions from smallest to biggest is: multicrystalline (multi-), monocrystalline (mono-), amorphous (amor-) crystalline silicon cells. The multi- cell characteristics are less susceptible to light intensity and cell temperature. Secondly, the neural network (ANN) prediction results are very close to the experimental data, and are also influenced by numbers of hidden neurons. Thirdly, the relationship between prediction results and the number of hidden units is not a simple function. For multi- and amorphous crystalline cell, three or four hidden layer units resulted in high correlation coefficients and low MSEs. For mono-crystalline cell, the best results were achieved at the hidden layer unit of 8.

## Supporting information

S1 Data *I-V* and *P-V* curves of mono-crystalline, multi-crystalline and amorphous crystalline silicon PV cells measured at two extreme conditions. To confirm the effects of light intensity and temperature on PV cells electrical characteristics, the *I-V* and *P-V* curves of mono-crystalline, multi-crystalline and amorphous crystalline silicon PV cells measured at two extreme conditions: the lowest light intensity and temperature (the 1st tranche (light intensity) and -10°C (temperature)), as well as the highest light intensity and temperature (6th tranche and 40°C) was given.
(DOC)

S2 Data The measured output max power of mono-crystalline, multi-crystalline and amorphous crystalline silicon PV cells at different conditions. The experimental dataset of output max power of mono-crystalline, multi-crystalline and amorphous crystalline silicon PV cells under different conditions were given. Those experimental data points were used to train the neuron network and to validate the prediction results.
(DOC)
