# Supplementary material for: A neural network based computational model to predict the output power of different types of photovoltaic cells
Source: PLoS One. 2017 Sep 12;12(9):e0184561. doi: 10.1371/journal.pone.0184561 (PMC5595326; doi:10.1371/journal.pone.0184561)
Supplement: S1 Data — To confirm the effects of light intensity and temperature on PV cells electrical characteristics, the I-V and P-V curves of mono-crystalline, multi-crystalline and amorphous crystalline silicon PV cells measured at two extreme conditions: the lowest light intensity and temperature (the 1st tranche (light intensity) and -10°C (temperature)), as well as the highest light intensity and temperature (6th tranche and 40°C) was given. (DOC) [file pone.0184561.s001.doc]

*I-V* and *P-V* data of mono-crystalline at the 1st tranche (light intensity) and -10 ^o^C (temperature)

| V(V) | I(mA) | P(mW) |
| --- | --- | --- |
| 0.006 | 12.526 | 0.07516 |
| 0.055 | 12.505 | 0.68778 |
| 0.113 | 12.523 | 1.4151 |
| 0.169 | 12.488 | 2.11047 |
| 0.228 | 12.476 | 2.84453 |
| 0.285 | 12.46 | 3.5511 |
| 0.343 | 12.505 | 4.28922 |
| 0.397 | 12.466 | 4.949 |
| 0.455 | 12.441 | 5.66066 |
| 0.513 | 12.423 | 6.373 |
| 0.574 | 12.415 | 7.12621 |
| 0.63 | 12.41 | 7.8183 |
| 0.689 | 12.401 | 8.54429 |
| 0.744 | 12.397 | 9.22337 |
| 0.804 | 12.327 | 9.91091 |
| 0.861 | 12.381 | 10.66004 |
| 0.92 | 12.353 | 11.36476 |
| 0.976 | 12.323 | 12.02725 |
| 1.035 | 12.254 | 12.68289 |
| 1.091 | 12.212 | 13.32329 |
| 1.15 | 12.261 | 14.10015 |
| 1.205 | 12.253 | 14.76487 |
| 1.265 | 12.251 | 15.49752 |
| 1.32 | 12.26 | 16.1832 |
| 1.377 | 12.246 | 16.86274 |
| 1.435 | 12.265 | 17.60028 |
| 1.496 | 12.283 | 18.37537 |
| 1.553 | 12.315 | 19.12519 |
| 1.608 | 12.26 | 19.71408 |
| 1.665 | 12.249 | 20.39459 |
| 1.722 | 12.265 | 21.12033 |
| 1.784 | 12.285 | 21.91644 |
| 1.838 | 12.27 | 22.55226 |
| 1.899 | 12.241 | 23.24566 |
| 1.954 | 12.224 | 23.8857 |
| 2.014 | 12.223 | 24.61712 |
| 2.071 | 12.199 | 25.26413 |
| 2.13 | 12.195 | 25.97535 |
| 2.186 | 12.145 | 26.54897 |
| 2.245 | 12.112 | 27.19144 |
| 2.302 | 12.059 | 27.75982 |
| 2.36 | 11.91 | 28.1076 |
| 2.417 | 11.756 | 28.41425 |
| 2.475 | 11.572 | 28.6407 |
| 2.532 | 11.294 | 28.59641 |
| 2.587 | 10.988 | 28.42596 |
| 2.648 | 10.599 | 28.06615 |
| 2.702 | 10.108 | 27.31182 |
| 2.764 | 9.435 | 26.07834 |
| 2.82 | 8.705 | 24.5481 |
| 2.879 | 7.714 | 22.20861 |
| 2.935 | 6.539 | 19.19197 |
| 2.991 | 5.142 | 15.37972 |
| 3.048 | 3.485 | 10.62228 |
| 3.109 | 1.443 | 4.48629 |
| 3.146 | 0 | 0 |

*I-V* and *P-V* data of mono-crystalline at the 6th tranche and 40 ^o^C

| V(V) | I(mA) | P(mW) |
| --- | --- | --- |
| 0.007 | 28.37 | 0.19859 |
| 0.062 | 28.34 | 1.75708 |
| 0.109 | 28.31 | 3.08579 |
| 0.159 | 28.3 | 4.4997 |
| 0.208 | 28.26 | 5.87808 |
| 0.258 | 28.22 | 7.28076 |
| 0.307 | 28.22 | 8.66354 |
| 0.355 | 28.2 | 10.011 |
| 0.405 | 28.13 | 11.39265 |
| 0.455 | 28.17 | 12.81735 |
| 0.504 | 28.14 | 14.18256 |
| 0.553 | 28.12 | 15.55036 |
| 0.602 | 28.12 | 16.92824 |
| 0.652 | 28.08 | 18.30816 |
| 0.7 | 28.06 | 19.642 |
| 0.75 | 28.05 | 21.0375 |
| 0.799 | 27.98 | 22.35602 |
| 0.849 | 27.99 | 23.76351 |
| 0.898 | 28.01 | 25.15298 |
| 0.946 | 28 | 26.488 |
| 0.996 | 27.96 | 27.84816 |
| 1.045 | 27.94 | 29.1973 |
| 1.096 | 27.96 | 30.64416 |
| 1.143 | 27.94 | 31.93542 |
| 1.193 | 27.9 | 33.2847 |
| 1.242 | 27.88 | 34.62696 |
| 1.292 | 27.91 | 36.05972 |
| 1.34 | 27.95 | 37.453 |
| 1.39 | 27.96 | 38.8644 |
| 1.439 | 27.89 | 40.13371 |
| 1.489 | 27.9 | 41.5431 |
| 1.538 | 27.87 | 42.86406 |
| 1.587 | 27.88 | 44.24556 |
| 1.636 | 27.86 | 45.57896 |
| 1.686 | 27.94 | 47.10684 |
| 1.735 | 27.89 | 48.38915 |
| 1.783 | 27.88 | 49.71004 |
| 1.833 | 27.84 | 51.03072 |
| 1.884 | 27.78 | 52.33752 |
| 1.932 | 27.74 | 53.59368 |
| 1.981 | 27.61 | 54.69541 |
| 2.029 | 27.39 | 55.57431 |
| 2.079 | 27.03 | 56.19537 |
| 2.13 | 26.61 | 56.6793 |
| 2.178 | 25.88 | 56.36664 |
| 2.227 | 25.05 | 55.78635 |
| 2.276 | 23.9 | 54.3964 |
| 2.327 | 22.32 | 51.93864 |
| 2.375 | 20.5 | 48.6875 |
| 2.424 | 18.23 | 44.18952 |
| 2.473 | 15.39 | 38.05947 |
| 2.524 | 11.78 | 29.73272 |
| 2.572 | 7.68 | 19.75296 |
| 2.621 | 2.87 | 7.52227 |
| 2.647 | 0 | 0 |

*I-V* and *P-V* data of multi-crystalline silicon PV cells measured at the 1st tranche (light intensity) and -10 ^o^C (temperature)

| V(V) | I(mA) | P(mW) |
| --- | --- | --- |
| 0.006 | 14.003 | 0.08402 |
| 0.052 | 13.956 | 0.72571 |
| 0.106 | 13.967 | 1.4805 |
| 0.16 | 13.947 | 2.23152 |
| 0.214 | 13.924 | 2.97974 |
| 0.269 | 13.934 | 3.74825 |
| 0.322 | 13.883 | 4.47033 |
| 0.377 | 13.878 | 5.23201 |
| 0.43 | 13.86 | 5.9598 |
| 0.485 | 13.824 | 6.70464 |
| 0.539 | 13.832 | 7.45545 |
| 0.593 | 13.73 | 8.14189 |
| 0.648 | 13.701 | 8.87825 |
| 0.702 | 13.722 | 9.63284 |
| 0.756 | 13.696 | 10.35418 |
| 0.81 | 13.678 | 11.07918 |
| 0.864 | 13.664 | 11.8057 |
| 0.919 | 13.625 | 12.52138 |
| 0.973 | 13.575 | 13.20848 |
| 1.027 | 13.598 | 13.96515 |
| 1.081 | 13.61 | 14.71241 |
| 1.135 | 13.655 | 15.49843 |
| 1.19 | 13.676 | 16.27444 |
| 1.244 | 13.618 | 16.94079 |
| 1.298 | 13.549 | 17.5866 |
| 1.352 | 13.609 | 18.39937 |
| 1.407 | 13.62 | 19.16334 |
| 1.462 | 13.627 | 19.92267 |
| 1.515 | 13.527 | 20.49341 |
| 1.569 | 13.505 | 21.18934 |
| 1.625 | 13.479 | 21.90338 |
| 1.678 | 13.521 | 22.68824 |
| 1.733 | 13.51 | 23.41283 |
| 1.786 | 13.512 | 24.13243 |
| 1.842 | 13.451 | 24.77674 |
| 1.895 | 13.42 | 25.4309 |
| 1.95 | 13.425 | 26.17875 |
| 2.003 | 13.401 | 26.8422 |
| 2.058 | 13.357 | 27.48871 |
| 2.112 | 13.383 | 28.2649 |
| 2.166 | 13.32 | 28.85112 |
| 2.22 | 13.226 | 29.36172 |
| 2.274 | 13.207 | 30.03272 |
| 2.329 | 13.172 | 30.67759 |
| 2.383 | 13.064 | 31.13151 |
| 2.437 | 12.801 | 31.19604 |
| 2.492 | 12.669 | 31.57115 |
| 2.546 | 12.41 | 31.59586 |
| 2.6 | 12.119 | 31.5094 |
| 2.654 | 11.681 | 31.00137 |
| 2.709 | 11.134 | 30.16201 |
| 2.763 | 10.439 | 28.84296 |
| 2.817 | 9.56 | 26.93052 |
| 2.871 | 8.491 | 24.37766 |
| 2.925 | 7.291 | 21.32618 |
| 2.98 | 5.789 | 17.25122 |
| 3.034 | 4.182 | 12.68819 |
| 3.088 | 2.321 | 7.16725 |
| 3.149 | 0 | 0 |

*I-V* and *P-V* data of multi-crystalline silicon PV cells measured at the 6th tranche and 40 ^o^C

| V(V) | I(mA) | P(mW) |
| --- | --- | --- |
| 0.007 | 30.96 | 0.21672 |
| 0.06 | 30.96 | 1.8576 |
| 0.105 | 30.9 | 3.2445 |
| 0.154 | 30.9 | 4.7586 |
| 0.201 | 30.74 | 6.17874 |
| 0.248 | 30.78 | 7.63344 |
| 0.295 | 30.7 | 9.0565 |
| 0.344 | 30.68 | 10.55392 |
| 0.39 | 30.6 | 11.934 |
| 0.439 | 30.5 | 13.3895 |
| 0.486 | 30.48 | 14.81328 |
| 0.534 | 30.4 | 16.2336 |
| 0.58 | 30.36 | 17.6088 |
| 0.628 | 30.32 | 19.04096 |
| 0.676 | 30.24 | 20.44224 |
| 0.723 | 30.23 | 21.85629 |
| 0.771 | 30.18 | 23.26878 |
| 0.818 | 30.13 | 24.64634 |
| 0.866 | 30.04 | 26.01464 |
| 0.912 | 30.02 | 27.37824 |
| 0.961 | 29.99 | 28.82039 |
| 1.007 | 29.92 | 30.12944 |
| 1.056 | 29.88 | 31.55328 |
| 1.102 | 29.82 | 32.86164 |
| 1.149 | 29.76 | 34.19424 |
| 1.198 | 29.71 | 35.59258 |
| 1.245 | 29.7 | 36.9765 |
| 1.293 | 29.63 | 38.31159 |
| 1.34 | 29.54 | 39.5836 |
| 1.388 | 29.52 | 40.97376 |
| 1.435 | 29.46 | 42.2751 |
| 1.483 | 29.48 | 43.71884 |
| 1.529 | 29.44 | 45.01376 |
| 1.578 | 29.4 | 46.3932 |
| 1.624 | 29.31 | 47.59944 |
| 1.673 | 29.27 | 48.96871 |
| 1.719 | 29.24 | 50.26356 |
| 1.766 | 29.23 | 51.62018 |
| 1.815 | 29.13 | 52.87095 |
| 1.861 | 28.97 | 53.91317 |
| 1.91 | 28.83 | 55.0653 |
| 1.956 | 28.5 | 55.746 |
| 2.005 | 28.18 | 56.5009 |
| 2.052 | 27.68 | 56.79936 |
| 2.1 | 26.92 | 56.532 |
| 2.147 | 26.12 | 56.07964 |
| 2.195 | 25 | 54.875 |
| 2.241 | 23.72 | 53.15652 |
| 2.289 | 22.15 | 50.70135 |
| 2.337 | 20.19 | 47.18403 |
| 2.384 | 18 | 42.912 |
| 2.432 | 15.4 | 37.4528 |
| 2.478 | 12.55 | 31.0989 |
| 2.527 | 9.24 | 23.34948 |
| 2.573 | 5.64 | 14.51172 |
| 2.622 | 1.58 | 4.14276 |
| 2.642 | 0 | 0 |

*I-V* and *P-V* data of amorphous crystalline silicon PV cells measured at the 1st tranche (light intensity) and -10 ^o^C (temperature)

| V(V) | I(mA) | P(mW) |
| --- | --- | --- |
| 0.006 | 2.449 | 0.01469 |
| 0.063 | 2.438 | 0.15359 |
| 0.111 | 2.437 | 0.27051 |
| 0.16 | 2.435 | 0.3896 |
| 0.21 | 2.434 | 0.51114 |
| 0.259 | 2.427 | 0.62859 |
| 0.309 | 2.42 | 0.74778 |
| 0.359 | 2.425 | 0.87058 |
| 0.41 | 2.41 | 0.9881 |
| 0.458 | 2.409 | 1.10332 |
| 0.508 | 2.397 | 1.21768 |
| 0.557 | 2.39 | 1.33123 |
| 0.607 | 2.387 | 1.44891 |
| 0.657 | 2.38 | 1.56366 |
| 0.706 | 2.372 | 1.67463 |
| 0.756 | 2.362 | 1.78567 |
| 0.807 | 2.351 | 1.89726 |
| 0.855 | 2.344 | 2.00412 |
| 0.905 | 2.33 | 2.10865 |
| 0.953 | 2.319 | 2.21001 |
| 1.005 | 2.308 | 2.31954 |
| 1.054 | 2.295 | 2.41893 |
| 1.103 | 2.276 | 2.51043 |
| 1.153 | 2.262 | 2.60809 |
| 1.203 | 2.245 | 2.70074 |
| 1.252 | 2.228 | 2.78946 |
| 1.302 | 2.208 | 2.87482 |
| 1.351 | 2.188 | 2.95599 |
| 1.401 | 2.17 | 3.04017 |
| 1.452 | 2.143 | 3.11164 |
| 1.5 | 2.124 | 3.186 |
| 1.55 | 2.103 | 3.25965 |
| 1.6 | 2.076 | 3.3216 |
| 1.649 | 2.057 | 3.39199 |
| 1.7 | 2.022 | 3.4374 |
| 1.748 | 1.996 | 3.48901 |
| 1.798 | 1.967 | 3.53667 |
| 1.849 | 1.932 | 3.57227 |
| 1.897 | 1.899 | 3.6024 |
| 1.947 | 1.855 | 3.61169 |
| 1.996 | 1.814 | 3.62074 |
| 2.046 | 1.774 | 3.6296 |
| 2.096 | 1.72 | 3.60512 |
| 2.145 | 1.661 | 3.56285 |
| 2.196 | 1.593 | 3.49823 |
| 2.245 | 1.511 | 3.3922 |
| 2.294 | 1.419 | 3.25519 |
| 2.345 | 1.31 | 3.07195 |
| 2.394 | 1.175 | 2.81295 |
| 2.443 | 1.026 | 2.50652 |
| 2.493 | 0.838 | 2.08913 |
| 2.542 | 0.637 | 1.61925 |
| 2.592 | 0.398 | 1.03162 |
| 2.642 | 0.132 | 0.34874 |
| 2.666 | 0 | 0 |

*I-V* and *P-V* data of amorphous crystalline silicon PV cells measured at the 6th tranche and 40 ^o^C

| V(V) | I(mA) | P(mW) |
| --- | --- | --- |
| 0.006 | 5.844 | 0.03506 |
| 0.054 | 5.826 | 0.3146 |
| 0.096 | 5.825 | 0.5592 |
| 0.137 | 5.832 | 0.79898 |
| 0.181 | 5.811 | 1.05179 |
| 0.222 | 5.795 | 1.28649 |
| 0.266 | 5.798 | 1.54227 |
| 0.309 | 5.78 | 1.78602 |
| 0.35 | 5.771 | 2.01985 |
| 0.394 | 5.762 | 2.27023 |
| 0.435 | 5.752 | 2.50212 |
| 0.479 | 5.753 | 2.75569 |
| 0.521 | 5.74 | 2.99054 |
| 0.563 | 5.728 | 3.22486 |
| 0.607 | 5.716 | 3.46961 |
| 0.648 | 5.706 | 3.69749 |
| 0.691 | 5.689 | 3.9311 |
| 0.734 | 5.675 | 4.16545 |
| 0.776 | 5.663 | 4.39449 |
| 0.818 | 5.655 | 4.62579 |
| 0.862 | 5.642 | 4.8634 |
| 0.904 | 5.623 | 5.08319 |
| 0.947 | 5.599 | 5.30225 |
| 0.989 | 5.593 | 5.53148 |
| 1.031 | 5.563 | 5.73545 |
| 1.075 | 5.547 | 5.96302 |
| 1.117 | 5.53 | 6.17701 |
| 1.159 | 5.508 | 6.38377 |
| 1.203 | 5.485 | 6.59846 |
| 1.244 | 5.459 | 6.791 |
| 1.288 | 5.421 | 6.98225 |
| 1.33 | 5.382 | 7.15806 |
| 1.372 | 5.355 | 7.34706 |
| 1.416 | 5.297 | 7.50055 |
| 1.458 | 5.239 | 7.63846 |
| 1.5 | 5.185 | 7.7775 |
| 1.542 | 5.108 | 7.87654 |
| 1.585 | 5.02 | 7.9567 |
| 1.628 | 4.931 | 8.02767 |
| 1.67 | 4.819 | 8.04773 |
| 1.714 | 4.694 | 8.04552 |
| 1.755 | 4.545 | 7.97648 |
| 1.799 | 4.361 | 7.84544 |
| 1.84 | 4.172 | 7.67648 |
| 1.883 | 3.944 | 7.42655 |
| 1.926 | 3.678 | 7.08383 |
| 1.968 | 3.406 | 6.70301 |
| 2.012 | 3.078 | 6.19294 |
| 2.054 | 2.751 | 5.65055 |
| 2.096 | 2.369 | 4.96542 |
| 2.139 | 1.953 | 4.17747 |
| 2.181 | 1.515 | 3.30422 |
| 2.224 | 1.048 | 2.33075 |
| 2.266 | 0.555 | 1.25763 |
| 2.309 | 0.029 | 0.06696 |
| 2.313 | 0 | 0 |
