# Supplementary material for: A neural network based computational model to predict the output power of different types of photovoltaic cells
Source: PLoS One. 2017 Sep 12;12(9):e0184561. doi: 10.1371/journal.pone.0184561 (PMC5595326; doi:10.1371/journal.pone.0184561)
Supplement: S2 Data — The experimental dataset of output max power of mono-crystalline, multi-crystalline and amorphous crystalline silicon PV cells under different conditions were given. Those experimental data points were used to train the neuron network and to validate the prediction results. (DOC) [file pone.0184561.s002.doc]

the measured maximum output power of mono-cells under different light intensity and temperature

| light intensity (W/m2) | temperature(OC) | maximum output power(mW) |
| --- | --- | --- |
| 1100 | -10 | 63.45648 |
| 1100 | -5 | 62.3604 |
| 1100 | 0 | 61.04862 |
| 1100 | 5 | 60.64896 |
| 1100 | 10 | 61.32842 |
| 1100 | 15 | 61.5802 |
| 1100 | 20 | 61.75558 |
| 1100 | 25 | 60.56505 |
| 1100 | 27 | 60.11885 |
| 1100 | 35 | 58.344 |
| 1100 | 30 | 59.29581 |
| 1100 | 40 | 56.6793 |
| 1000 | 40 | 50.07528 |
| 1000 | 35 | 51.55156 |
| 1000 | 30 | 52.38296 |
| 1000 | 27 | 54.71111 |
| 1000 | 25 | 54.90802 |
| 1000 | 20 | 56.0598 |
| 1000 | 15 | 56.94192 |
| 1000 | 10 | 56.68996 |
| 1000 | 5 | 55.16802 |
| 1000 | 0 | 55.21923 |
| 1000 | -5 | 56.63636 |
| 1000 | -10 | 56.0556 |
| 900 | -10 | 50.9568 |
| 900 | -5 | 50.26164 |
| 900 | 0 | 49.3248 |
| 900 | 5 | 48.30408 |
| 900 | 10 | 41.74317 |
| 900 | 20 | 49.66968 |
| 900 | 15 | 48.76704 |
| 900 | 25 | 49.66232 |
| 900 | 27 | 49.30632 |
| 900 | 30 | 58.51127 |
| 900 | 35 | 57.20399 |
| 900 | 40 | 55.49819 |
| 800 | 40 | 39.976 |
| 800 | 35 | 41.18928 |
| 800 | 30 | 42.37116 |
| 800 | 27 | 42.89934 |
| 800 | 25 | 43.26718 |
| 800 | 20 | 44.05774 |
| 800 | 15 | 43.72221 |
| 800 | 10 | 45.01872 |
| 800 | 5 | 44.38962 |
| 800 | 0 | 44.26902 |
| 800 | -5 | 44.29503 |
| 800 | -10 | 44.53412 |
| 700 | -10 | 37.9104 |
| 700 | -5 | 37.88042 |
| 700 | 0 | 38.9873 |
| 700 | 5 | 37.51434 |
| 700 | 10 | 37.16724 |
| 700 | 15 | 38.13875 |
| 700 | 20 | 37.39125 |
| 700 | 25 | 36.6663 |
| 700 | 27 | 36.51375 |
| 700 | 30 | 35.89926 |
| 700 | 35 | 35.0244 |
| 700 | 40 | 34.0734 |
| 600 | 40 | 26.59646 |
| 600 | 35 | 27.43013 |
| 600 | 30 | 28.1177 |
| 600 | 27 | 28.82666 |
| 600 | 25 | 28.86498 |
| 600 | 20 | 29.64813 |
| 600 | 15 | 29.95995 |
| 600 | 10 | 30.47161 |
| 600 | 5 | 28.77417 |
| 600 | -10 | 28.6407 |
| 600 | 0 | 28.52005 |
| 600 | -5 | 28.28462 |

the measured maximum output power of multi-cells under different light intensity and temperature

| light intensity (W/m2) | temperature(OC) | maximum output power(mW) |
| --- | --- | --- |
| 1100 | 40 | 56.79936 |
| 1100 | 35 | 59.20814 |
| 1100 | 30 | 60.77448 |
| 1100 | 27 | 61.9692 |
| 1100 | 25 | 62.27501 |
| 1100 | 20 | 63.51532 |
| 1100 | 15 | 64.79983 |
| 1100 | 10 | 63.34613 |
| 1100 | 5 | 64.36288 |
| 1100 | 0 | 64.35 |
| 1100 | -5 | 64.778 |
| 1100 | -10 | 65.09118 |
| 1000 | -10 | 57.76876 |
| 1000 | -5 | 56.55516 |
| 1000 | 0 | 58.14914 |
| 1000 | 5 | 59.21775 |
| 1000 | 10 | 59.92508 |
| 1000 | 15 | 58.41048 |
| 1000 | 20 | 57.43584 |
| 1000 | 27 | 55.54716 |
| 1000 | 25 | 56.0119 |
| 1000 | 30 | 54.84625 |
| 1000 | 35 | 53.4843 |
| 1000 | 40 | 52.23761 |
| 900 | 40 | 48.216 |
| 900 | 35 | 49.335 |
| 900 | 30 | 50.48888 |
| 900 | 27 | 51.29936 |
| 900 | 25 | 51.91056 |
| 900 | 20 | 53.016 |
| 900 | 15 | 53.44493 |
| 900 | 10 | 54.87384 |
| 900 | 5 | 55.37178 |
| 900 | 0 | 53.17 |
| 900 | -5 | 53.1262 |
| 900 | -10 | 53.0688 |
| 800 | -10 | 45.66128 |
| 800 | -5 | 46.2726 |
| 800 | 0 | 46.13896 |
| 800 | 10 | 45.52726 |
| 800 | 5 | 45.7595 |
| 800 | 15 | 45.24849 |
| 800 | 20 | 44.6592 |
| 800 | 25 | 43.99164 |
| 800 | 27 | 43.83708 |
| 800 | 30 | 43.1824 |
| 800 | 35 | 42.2136 |
| 800 | 40 | 41.15616 |
| 700 | 40 | 35.49 |
| 700 | 35 | 36.63222 |
| 700 | 30 | 37.16135 |
| 700 | 27 | 37.86738 |
| 700 | 25 | 38.33466 |
| 700 | 20 | 39.23205 |
| 700 | 15 | 40.06428 |
| 700 | 10 | 40.55168 |
| 700 | 5 | 39.80618 |
| 700 | 0 | 40.2268 |
| 700 | -5 | 40.196 |
| 700 | -10 | 40.67416 |
| 600 | -10 | 31.59586 |
| 600 | 0 | 31.55428 |
| 600 | -5 | 30.97671 |
| 600 | 5 | 30.99092 |
| 600 | 10 | 30.48011 |
| 600 | 15 | 30.54776 |
| 600 | 20 | 30.24175 |
| 600 | 25 | 31.45526 |
| 600 | 27 | 30.9686 |
| 600 | 30 | 30.63554 |
| 600 | 35 | 29.8356 |
| 600 | 40 | 28.7956 |

the measured maximum output power of amorphous crystalline silicon PV cells under different light intensity and temperature

| light intensity (W/m2) | temperature(OC) | maximum output power(mW) |
| --- | --- | --- |
| 1100 | 40 | 8.04773 |
| 1100 | 35 | 8.19398 |
| 1100 | 30 | 8.28986 |
| 1100 | 27 | 8.15797 |
| 1100 | 25 | 8.31659 |
| 1100 | 20 | 8.39507 |
| 1100 | 15 | 8.27304 |
| 1100 | 10 | 8.0767 |
| 1100 | 5 | 7.906 |
| 1100 | 0 | 7.7814 |
| 1100 | -5 | 7.75257 |
| 1100 | -10 | 7.64408 |
| 1000 | 40 | 6.79438 |
| 1000 | 35 | 7.03922 |
| 1000 | 30 | 7.10644 |
| 1000 | 27 | 7.15806 |
| 1000 | 25 | 7.24771 |
| 1000 | 20 | 7.33017 |
| 1000 | 15 | 7.38882 |
| 1000 | 10 | 7.19328 |
| 1000 | 5 | 7.0589 |
| 1000 | 0 | 6.95735 |
| 1000 | -5 | 6.88517 |
| 1000 | -10 | 6.80247 |
| 900 | -10 | 6.14955 |
| 900 | -5 | 6.19745 |
| 900 | 0 | 6.39277 |
| 900 | 5 | 6.58186 |
| 900 | 10 | 6.70824 |
| 900 | 15 | 6.67024 |
| 900 | 20 | 6.51745 |
| 900 | 25 | 6.40276 |
| 900 | 27 | 6.36411 |
| 900 | 30 | 6.29082 |
| 900 | 35 | 6.13548 |
| 900 | 40 | 6.06273 |
| 800 | 40 | 5.22275 |
| 800 | 35 | 5.33172 |
| 800 | 30 | 5.46516 |
| 800 | 27 | 5.53347 |
| 800 | 25 | 5.59027 |
| 800 | 10 | 5.54206 |
| 800 | 5 | 5.47307 |
| 800 | 20 | 5.66307 |
| 800 | 15 | 5.69718 |
| 800 | 0 | 5.42407 |
| 800 | -5 | 5.36585 |
| 800 | -10 | 5.29158 |
| 700 | -10 | 4.54132 |
| 700 | -5 | 4.59928 |
| 700 | 0 | 4.66231 |
| 700 | 5 | 4.67382 |
| 700 | 10 | 4.72649 |
| 700 | 15 | 4.758 |
| 700 | 20 | 4.7304 |
| 700 | 25 | 4.63009 |
| 700 | 27 | 4.60411 |
| 700 | 30 | 4.45795 |
| 700 | 35 | 4.39469 |
| 700 | 40 | 4.108 |
| 600 | -10 | 3.6296 |
| 600 | 0 | 4.91316 |
| 600 | -5 | 3.66367 |
| 600 | 5 | 4.99962 |
| 600 | 10 | 5.09185 |
| 600 | 15 | 5.14228 |
| 600 | 20 | 5.05791 |
| 600 | 25 | 5.03375 |
| 600 | 27 | 5.00103 |
| 600 | 30 | 4.95258 |
| 600 | 35 | 4.86837 |
| 600 | 40 | 4.82433 |
